# 25 years of CIC – achievements and future goals

**DOI:** 10.1186/1758-2946-4-S1-O1

**Published:** 2012-05-01

**Authors:** Johann Gasteiger

**Affiliations:** 1 Computer-Chemie-Centrum, University of Erlangen Nuremberg, Germany; 2 Molecular Networks GmbH, Henkestr. 91, D-91052 Erlangen, Germany

## 

25 years ago several persons within the German Chemical Society realized that computer methods will increasingly play a major role in the processing of chemical information. This led to the foundation of the Division “Chemie-Information-Computer (CIC)” and the establishment of a Workshop “Software-Entwicklung in der Chemie”, Parallel to this meeting the “Molecular Modeling Workshop” was initiated covering more the fields of molecular modeling and drug design. Over the years the CIC Workshop embraced the English language and metamorphosed into a European endeavor.

From the very beginning scientists of all disciplines of chemistry participated in this Workshop. Initially, a major emphasis was put on the building of databases paralleling the work on the Beilstein, the Gmelin, the SpecInfo and the ChemInform databases. However, already in the early phases of this Workshop research was presented on converting chemical information into knowledge and using this knowledge in systems for automatic structure elucidation or the design of organic syntheses.

The Workshop certainly was instrumental in introducing Chemoinformatics in Germany. Many problems in Chemoinformatics were tackled and solved. However, it must be realized that by far not all problems have been solved. Furthermore, the recognition of Chemoinformatics as a scientific discipline still leaves something to be desired. These topics will be discussed and approaches for their improvement will be outlined.

